# Mass Spectrometry-Based Metabolomics to Elucidate Functions in Marine Organisms and Ecosystems

**DOI:** 10.3390/md10040849

**Published:** 2012-04-05

**Authors:** Sophie Goulitquer, Philippe Potin, Thierry Tonon

**Affiliations:** 1 Plate-forme MetaboMER, CNRS & UPMC, FR2424, Station Biologique, 29680 Roscoff, France; 2 UMR 7139 Marine Plants and Biomolecules, UPMC Univ Paris 6, Station Biologique, 29680 Roscoff, France; Email: potin@sb-roscoff.fr (P.P.); tonon@sb-roscoff.fr (T.T.); 3 UMR 7139 Marine Plants and Biomolecules, CNRS, Station Biologique, 29680 Roscoff, France

**Keywords:** metabolomics, mass spectrometry, LC-MS, GC-MS, targeted and untargeted profiling, systems biology, chemical ecology, databases

## Abstract

Marine systems are very diverse and recognized as being sources of a wide range of biomolecules. This review provides an overview of metabolite profiling based on mass spectrometry (MS) approaches in marine organisms and their environments, focusing on recent advances in the field. We also point out some of the technical challenges that need to be overcome in order to increase applications of metabolomics in marine systems, including extraction of chemical compounds from different matrices and data management. Metabolites being important links between genotype and phenotype, we describe added value provided by integration of data from metabolite profiling with other layers of omics, as well as their importance for the development of systems biology approaches in marine systems to study several biological processes, and to analyze interactions between organisms within communities. The growing importance of MS-based metabolomics in chemical ecology studies in marine ecosystems is also illustrated.

## Abbreviations

AMDIS: Automated mass spectral deconvolution and identification system
AMS: Aerosol mass spectrometry
APCI: Atmospheric pressure chemical ionization
ARA: arachidonic acid
CCAP: Culture collection of algae and protozoa
DAD: Diode array detector
DESI: Desorption electrospray ionization
DHA: Docosahexaenoic acid
ECNI: Electron capture negative ionization
ELSD: Evaporative light scattering detector
EI: Electronic impact
FWS: *Ectocarpus* freshwater strain
GC: Gas chromatography
HS: Headspace
I3: Integrated Infrastructure Initiative
ICP: Inductively coupled plasma
FP7: Framework Programme 7
FT-ICR: Fourier transform ion cyclotron resonance
LAESI: Laser ablation electrospray ionization
LC: Liquid chromatography
MALDI: Matrix assisted laser desorption ionization
MRM: Multiple reaction monitoring
MS: Mass spectrometry
NGS: Next Generation Sequencing
NICI: Negative ion chemical ionization
NMR: Nuclear magnetic resonance
PDMS: Polydimethylsiloxane
PUAs: Polyunsaturated aldehydes
PUFAs: Polyunsatured fatty acids
Q: quadrupole
SBSE: Stir bar sorptive extraction
SIMS: Secondary ion mass spectrometry
SPE: Solid phase extraction
SPME: Solid phase microextraction
SWS: *Ectocarpus* seawater strain
TD: Thermodesorption
TMS: Trimethylsilyl derivatives
TOF: Time of flight
UPLC: Ultra performance liquid chromatography
VHOCs: Volatile halogenated organic compounds
VOCs: Volatile organic compounds.

## 1. Introduction

Seas and oceans cover more than 70% of the surface of the globe. Marine environments are rather diverse, some of them being either stable or unstable, and this heterogeneity is related, to some extent, to distinct physico-chemical conditions like salinity, UV radiation, temperature, and pressure. Such habitats are occupied by a great diversity of prokaryotic and eukaryotic organisms, autotrophic or heterotrophic, accounting for about 2.2 million species of microbes, algae, plants, and animals according to recent estimations [1]. This number is likely to increase in the near future with many species awaiting description. Moreover, marine biological systems exhibit different levels of complexity, both at the level of single species and of populations.

One feature supporting this biodiversity is contained inside each individual, in the genetic information. Marine organisms possess an extraordinary variety of—often unique—structures related to different biological processes such as metabolic pathways, reproductive systems, development patterns, and sensory and defense mechanisms. Over the last decade, marine biologists have been extremely active in applying genomics at both the organism and the ecosystem level, and the quantity of genomic resources on marine organisms is now becoming highly significant. These resources can be found in several databases with different focus, such as Megx, CAMERA JGI, and GOLD (Table 1). Availability of these genomic data allows gauging the great molecular potential featured by these organisms, in particular for the production of a plethora of chemicals. Among these compounds, metabolites are small molecules produced mostly, but not exclusively, by enzymatic reactions, and represent one element of the phenotype. These intracellular and extracellular molecules can play important roles in many biological processes such as metabolism, reproduction, development, and response to changes in the environment (abiotic or biotic factors), at the level of organisms and/or populations. The complete set of these small molecules is called the metabolome. Its composition is altered by changes in gene expression and regulation of protein functions, and is thus under the influence of mutation(s) and effectors (endogenous or exogenous). Moreover, some metabolites can, in return, induce perturbations at the transcriptional level, and modify activity of proteins. Therefore, the metabolome has to be considered as a snapshot of an organism at a given time, and can be altered according to different life stages and growth conditions. Its characterization is important to better understand cellular systems, and to decode functions of genes. It is also likely that knowledge of the complex patterns of chemicals released by marine species will result in a paradigm shift, revealing new mechanisms for processes such as community functions, food location, or complex defensive and allelopathic interactions.

There is a long tradition to explore marine diversity in order to discover new molecules and identify bioactive compounds, since marine organisms produce a wide array of chemicals [2,3]. However, if this currently constitutes the rationale behind most of analyses carried out on these organisms, there is surely a shift from cataloging metabolites to asking broader biological questions about how metabolites reflect and affect cell function, and to integrate these results with data produced at different levels of cell organization to better understand marine (eco)systems. These changes are supported by the development of protocols and technologies that allow dealing with more samples at a time, more quickly, and for detection and quantification of an increasing complexity of compounds covering a very dynamic range of concentrations.

Measurement of metabolites is performed by metabolomics, a rather new aspect in the field of omics techniques, in particular when compared with approaches used to sequence genomes and to profile RNA and proteins. Nuclear magnetic resonance (NMR) and mass spectrometry (MS)-based techniques are among the most popular technologies available to perform medium to high throughput metabolite profiling, and have already been used extensively, for instance for phenotyping of several benchmark biological models, or for investigating interactions of living organisms with their environment (environmental metabolomics) [4,5,6]. The most common techniques to separate molecules before identification are gas chromatography (GC) and liquid chromatography (LC), even if other techniques have been developed. Efficient separation techniques, coupled with high-resolution MS, are appropriate to deal with high numbers of samples that need to be handled simultaneously and in shortest time, and that have to be combined to produce extended subsets of metabolites profiles, both for quantification and identification. An important aspect representing a challenging issue for metabolomics analysis of marine organisms is the high number of compounds with unknown structures. One way to overcome this shortcoming is to combine NMR and MS [7]. Interestingly, many metabolites involved in primary metabolism, in comparison with transcripts or proteins, are not so organism specific; thus, when analytical procedures are successfully applied for their measurement, these protocols can be transferred for determination of the same metabolites in other organisms [8]. 

**Table 1:** Database links.

**Mass Spectral Databases**		
	HMDB (Human Metabolome Database)	www.hmdb.ca/
	Metlin	http://metlin.scripps.edu/
	KNApSAcK	http://kanaya.naist.jp/KNApSAcK/
	MassBank	www.massbank.jp/
	GMD (Golm Metabolome Database)	http://gmd.mpimp-golm.mpg.de/
	FiehnLib (Fiehn Metabolome library)	http://fiehnlab.ucdavis.edu/projects/FiehnLib/index_html
	NIST	www.nist.gov/index.html
	MMCD	http://mmcd.nmrfam.wisc.edu/
**Metabolite Databases**		
	SWMD (Seaweed Metabolite Database)	www.swmd.co.in/
	ChEBI	www.ebi.ac.uk/chebi/
	DrugBank	www.drugbank.ca/
	PubChem	http://pubchem.ncbi.nlm.nih.gov/
	MarinLit	www.chem.canterbury.ac.nz/marinlit/marinlit.shtml
	LIPID MAPS	www.lipidmaps.org/
	Chemspider	www.chemspider.com/
	KEGG	www.genome.jp/kegg/
	MMCD	http://mmcd.nmrfam.wisc.edu/
**(Meta)Genomic Databases**		
	Megx	www.megx.net/
	CAMERA	http://camera.calit2.net/
	JGI	http://genome.jgi-psf.org/
	GOLD	www.genomesonline.org/cgi-bin/GOLD/index.cgi
Data Processing Links		
	XCMS	http://metlin.scripps.edu/xcms/
	MetAlign	www.pri.wur.nl/UK/products/MetAlign/
	MZmine2	http://mzmine.sourceforge.net/
	MetaboAnalyst	www.metaboanalyst.ca/MetaboAnalyst/
	metaP-Server	http://metabolomics.helmholtz-muenchen.de/metap2/
	MetAtt	http://metatt.metabolomics.ca/MetATT
	Metabolome Express	www.metabolome-express.org
	AMDIS	www.amdis.net/
	SpectConnect	http://spectconnect.mit.edu/
**National and International Research Networks**		
	ASSEMBLE	www.assemblemarine.org
	Biogenouest^®^	www.ouest-genopole.org
	EMBRC	www.EMBRC.eu

The introduction of state-of-the-art genomic approaches to marine biology has stimulated ground-breaking technological and theoretical advances, leading to the development of major new avenues of research. Recent technical developments in the field of metabolomics allow forecasting comprehensive metabolomics analyses in marine systems [9]. Data produced in different biological contexts will be useful to complement results obtained by other omics approaches (genomics, transcriptomics, and proteomics), in order to study physiological and environmental processes at different levels of organization. This will also permit to get insights on regulation and interactions at systems level in marine environments, these systems being single organisms or a community [10,11,12]. At the cellular level, this is particularly feasible for marine model organisms for which omics techniques are developed, and which represents different evolutionary lineages. In addition, since marine ecosystems feature significantly more biodiversity (at the phylum level) than terrestrial environments, comparative and functional genomics are driving marine organisms to the forefront of developmental and evolutionary biology. Moreover, environmental marine genomics opens new opportunities to understand the fundamentals of life, by studying diverse and numerous organisms, populations, and communities. Marine genomics knowledge also has direct applications in the management of natural and cultured resources, the protection of marine environments, and in marine (“blue”) biotechnology.

In this review, we attempt to illustrate the diversity of analyses conducted so far by targeted and untargeted MS-based profiling approaches in marine (eco)systems. To achieve this aim, we focus on recently published advances in the field.

## 2. Overview of Mass Spectrometry (MS)-Based Metabolic Profiling/Metabolomics in Marine Organisms

### 2.1. Bacteria (Heterotrophic and Cyanobacteria)

Bacteria have so far been the most extensively studied marine organisms for metabolite profiling. Use of ^13^C labeling techniques with GC-MS analysis [13] revealed important features of the central carbon metabolism in two members of the marine *Roseobacter clade* (Alphaproteobacteria). Additionally, several reports have been published on *Saccharophagus degradans*, a Gammaproteobacteria able to degrade complex macroalgal and land plant polysaccharides [14,15,16]. In particular, a GC-Time of flight (TOF) approach has been used to study metabolite profiles after culture in presence of different carbon sources, including agarose, galactose, glucose cellulose, xylose or xylan. Moreover, an in-house metabolic database named BinBase [17] served for metabolite identification.

Fractions of the metabolite content of other bacteria have been reported by targeted analysis. Phenazines, which are of special interest due to their antimicrobial activities, were investigated in bacteria affiliated to* Firmicutes, Alpha* and *Gammaproteobacteria*, and *Actinobacteria* by an LC-UV approach coupled to MS [18]. This study highlights the lack of a marine compounds database, as only 14 of 26 detected metabolites were identified. *Gammaproteobacteria* have been among the most common heterotrophic bacteria analyzed by metabolite profiling. For instance, through the characterization of a new strain within the species *Zooshikella*, application of LC-MS-MS revealed the presence of major metabolic compounds such as the red pigments prodigiosins and cycloprodigiosins [19]. In addition, multivariate statistical analyses of these global secondary metabolite profiling data led to the distinction of the new isolate from two closely related strains. In a similar way, high performance liquid chromatography (HPLC)-UV coupled with MS was used to profile secondary metabolites in pigmented and non-pigmented *Pseudoalteromonas* strains, in order to both determine if some of these metabolites could be used as biomarkers, and if some of them exhibited antibacterial activity [20]. Moreover, *Vibrionaceae*, which are widespread in the marine environment, have recently attracted interest for metabolite profiling, leading to the discovery of a huge amount of bioactive secondary metabolites based on LC-MS approach [21,22].

Among the Bacteroidetes, *Salinibacter ruber*, a halophilic bacterium, was incubated under different stress conditions before extraction of metabolites for analysis by direct infusion high-field ion cyclotron Fourier transform mass spectrometry (FT-ICR-MS), a quite unusual analytical method for metabolomics studies [23]. Despite the annotation for only a low number of the masses detected, the method allowed the identification of a number of changes associated to fatty acid metabolism, including glycerolipids and glycerophospholipids, under the experimental conditions tested.

Alongside heterotrophic bacteria, cyanobacteria are key players in aquatic environments. Most of the metabolomic MS-based studies for this type of bacteria focus on *Synechocystis *sp. PCC 6803, a freshwater strain [24,25,26]. However, Baran *et al.* [27] described an untargeted GC-MS method to estimate the breadth of metabolite uptake and release by *Synechococcus *sp. PCC 7002. In 2008, Esquenazi *et al.* [28] reported a method to visualize spatial distribution of secondary metabolites produced by several cyanobacteria (*Lyngbya majuscula *3L and JHB, *Oscillatoria nigro-viridis*, *Lyngbya bouillonii*, and a *Phormidium* species), using the natural product matrix assisted laser desorption ionization (MALDI)-TOF-imaging (npMALDI-I) approach. Further publication on this topic highlights the powerful combination of stable isotope feeding with MALDI-TOF imaging techniques to analyze the turnover rate of multiple natural products in the genus *Lyngbia*, and also to study more carefully the production of important chemicals such as jamaicamides [29]. In addition, to obtain a clearer understanding of the remarkable chemical diversity exhibited by this bacterial genus, occurrence of biosynthetic pathways for secondary metabolites in different strains was compared by LC-ESI-MS-MS (LC-ElectroSpray Ionization-MS-MS) and MALDI-TOF approaches [30]. Moreover, a recent study focused on the detection and identification of halogenated natural products from several distinct strains of *Lyngbya* [31].

### 2.2. Micro and Macroalgae

For many years, chemical profiling of marine algal extracts was restricted to targeted identification and quantification of selected classes of compounds relevant in the context of economical uses and/or of ecophysiology of these organisms. Aqueous extracts were particularly investigated to measure carbon metabolism-derived compounds such as sugar-nucleotides, polyols, storage and cell wall polysaccharides, using GC-flame ionization detector (FID), GC-MS, HPLC, or NMR methods. Other GC-based studies measured sterol compounds and free fatty acids that were further characterized when MS became more accessible for algal research. Pigment profiling was also very extensively used to describe the different taxa of algae, with major qualitative differences observed between the main algal lineages (red algae, green algae, Rhizaria, Dinoflagellates, Stramenopiles). These organic extracts are indeed very complex mixtures and have imposed major challenges for the analysts.

There are only a limited number of recent metabolomics analyses of microalgae that have been conducted using MS-based approaches, and most of them concern diatoms, this is why we focused on these organisms in the following section.

#### 2.2.1. Diatoms

Most of these data have been produced by GC-MS technology, due to its high sensitivity and robustness for targeted metabolites such as fatty acids, amino acids, oxylipins, or natural products. In 2009, Nappo *et al.* published a rather extensive description of *Cocconeis scutellum*, and identified more than 100 metabolites by GC-MS in both EI (Electronic Impact) and NICI (Negative Ion Chemical Ionization) modes [32]. In another study on *Skeletonema marinoi*, this NICI mode was preferred to detect and identify polyunsaturated aldehydes (PUAs) derivatized as pentafluorobenzyle-oximes [33] because of its high sensibility for halogenated compounds. The metabolome of this alga was further examined by GC-MS [34]. The derivatization process retained in this last study is currently used for plant metabolomics. First, methoximation is conducted with methoxyamine hydrochloride in pyridine, followed by trimethylsilylation with *N*-Methyl*-N-*trimethylsilyltrifluoroacetamide.

In addition to these rather broad analyses, more targeted studies have brought a lot of information on diatom metabolism. For instance, Lang *et al.* used a metabolite fingerprinting approach to establish chemotaxonomic markers by profiling more than 86 fatty acids in over 2000 strains of microalgae, including a number of diatoms, by GC-MS [35]. More recently, d’Ippolito *et al.* [36] combined different techniques, including tandem MS, to characterize oxylipins biosynthesis in the pennate diatom *Pseudo-nitzschia delicatissima*, and several polar ionic liquid stationary phases were tested in GC × GC analysis of fatty acids in *Cylindrotheca closterium* and *Seminavis robusta* [37]. In relation to these analyses, Yan *et al.* [38] profiled photosynthetic glycerolipids in three strains of *Skeletonema* by ultra performance liquid chromatography-quadrupole-time of flight (UPLC-Q-TOF). In a different context, Allen *et al.*, by combining GC-MS analysis of amino acids, organic acids and sugars with transcriptomic data, showed that the diatom ornithine-urea cycle represents a key pathway for anaplerotic carbon fixation into nitrogenous compounds that are essential for diatom growth [39].

To finish, it is noteworthy to mention that volatile halogenated compounds have also been detected in diatoms. Recently, cyanogen bromide (BrCN) has been demonstrated to control biofilm formation [40]. This metabolite has clearly been shown to be emitted by *Nitzschia cf pellucida*, thanks to a headspace-solid phase microextraction (SPME)-GC-MS approach.

#### 2.2.2. Macroalgae

In 2011, the first high coverage metabolomics study on red algae was published [41], and it described changes in metabolites produced by *Gracilaria vermiculophylla *in relation to defense response. Multivariate analysis of data acquired on both GC-MS and LC-MS systems, integrated with bioassays data, demonstrated that this invasive alga could deter herbivors by both quickly induced and slower activated chemical defense. Algae exposed to extracts produced from previously wounded algal tissue did not present statistically different LC-MS or GC-MS metabolite profiles from controls. However, in a distinct experiment, statistical analysis demonstrated that compounds derived from the arachidonic acid (ARA) metabolism were selectively induced and could be identified as biomarkers of chemical signalization after wounding.

Indeed, the wound response of the red alga *Gracilaria chilensis* was previously investigated using LC-MS targeted profiling of free fatty acid derivatives, and also pointed to an involvement of hydroxylated eicosanoids, in particular 8*R*-hydroxy eicosatetraenoic acid (8-HETE) and 7*S*,8*R*-dihydroxy eicosatetraenoic acid (7,8-di-HETE), as major players in the response [42]. Lipid metabolism of red algae other than *Gracilaria *spp*.* was also assessed by MS targeted analysis of oxylipins under various conditions. In response to methyl jasmonate or to the green algal pathogen *Acrochaete operculata*, the red alga *Chondrus crispus* produced oxidized derivatives of free fatty acids [43,44]. The presence of both C18 and C20 metabolites was highlighted by combining LC-MS and GC-MS methods. In a previous report, MS, combined with NMR, was also used to determine enzymatic activities such as a 5-LOX activity in *Rhodymenia pertusa* [45].

Metabolite profiling was not restricted to lipid metabolism in red algae, but applied also to several classes of secondary metabolites. Quantification of mycosporine-like amino acids (MAAs), compounds involved in stress responses in several types of marine photosynthetic organisms, has been performed in *Palmaria palmata* [46]. ESI-Trap MS analysis pointed out differences in MAAs contents of algae exposed to distinct levels of UV-radiation, despite absence of change in the oxygen radical absorbance capacity. These differences in MAAs contents were related to the variability in the anti-proliferative activities of *Palmaria*. In addition, desorption electrospray ionization mass spectrometry (DESI-MS) imaging, performed directly on the red alga *Callophycus serratus*, revealed that bromophycolides (brominated diterpene-benzoic acids) were found exclusively in association with distinct surface patches at concentrations sufficient for fungal inhibition [47]. DESI-MS also indicated the presence of these metabolites within internal algal tissue, and provided support for a role of these compounds as antifungal defenses. To complete these observations, a high diversity of secondary metabolites (at least 300) was reported for the *Portieria* species from the Philippines by GC-MS analysis, most of them remaining undescribed [48]. However, analysis of these data demonstrated extensive metabolite intra-specific variations between life-history stages within this species.

Halogenated compounds are well known in red and brown macroalgae, and their metabolism continues to attract interest, as illustrated by the recent publication of a comparative study of volatile halogenated compounds emitted by seaweeds [49]. Different MS approaches, such as ICP-MS (inductively coupled plasma), TOF-AMS (time-of-flight aerosol mass spectrometer), or TD-GC-MS (thermo-desorption), were combined to describe the whole halogenated metabolism in and around the six brown macroalgae and two red seaweeds tested. The latter showed a pattern in which iodomethane was abundant, whereas diiodomethane only played a minor role; opposite observations were made for brown algae. Effectively, these organisms are known for their capacity to accumulate and then to release halogens, in particular iodine, supporting their important contribution in the emission of volatile halogenated compounds in the marine boundary layer [50]. Secondary ion mass spectrometry (SIMS) has been used to analyze iodine distribution in *Laminaria digitata*. Chemical images revealed that iodine is mainly chelated as labile inorganic species by apoplastic macromolecules [51].

Brown algae are also considered as reservoirs for other types of secondary metabolites. For instance, Kledjus *et al.* suspected the presence of isoflavones, known as pharmaceutical products, in cyanobacteria and various red and brown macroalgae, because of the presence of precursors for this type of molecules in these organisms [52]. To check for the presence of isoflavones, these authors used ultra performance liquid chromatography (UPLC) coupled with MS. Determining fragmentation pathways, with a triple quadripole, allowed them to work in MRM (multiple reaction monitoring) mode and to reduce extraction steps. Eight isoflavone compounds were found for the first time in the different organisms tested, representing new direct evidence of presence of this type of molecule in these algae. Variable amounts of isoflavones, such as ononin, genistin, formonetin and biochanin, were determined in different algal samples, and these molecules were, for instance in brown algae, three times more abundant in *Sargassum muticum* than in *Sargassum vulgare*.

During the last decade, several methods to identify potential chemical markers of defense responses in brown macroalgae have been developed. For instance, hydrodistillation, focused microwave-assisted hydrodistillation, or supercritical fluid extraction, followed by GC/MS analysis, have been applied for extraction of volatile metabolites from *Dictyopteris membranacea* [53]. On the other hand, changes in fatty acids and their oxygenated derivatives in the kelp *L. digitata* have been monitored under stress conditions by LC-MS [54,55]. Targeted metabolite profiling was then continued in brown algae, and in particular in the model species *Ectocarpus siliculosus*, to analyze variations of some metabolites related to primary metabolism through the diurnal cycle [56], and under short-term non-lethal abiotic stress conditions [57]. In a recent review [58], Tonon et al. described various approaches to integrate metabolomic results with genomic and transcriptomic data to move towards systems biology approaches in the context of studying acclimation and adaptation processes to abiotic factors in *E. siliculosus*. Furthermore, this alga represents a good candidate to foresee fluxomic studies in the near future, for instance by incorporating ^13^C and ^15^N in the culture media, and by subsequently analyzing the produced metabolites by MS approaches to elucidate the metabolic pathways involved in these biological processes.

Most of the studies dealing with marine green macroalgae have so far focused on lipid metabolism. Variability of the fatty acid content within the genus *Codium* was investigated by GC-MS, and more than 40 volatile compounds, including dioic and fatty acids, were determined from two species [59]. Large variations in individual fatty acid content were observed according to species, location, and season. The same year, and to study the biosynthesis of PUAs in the marine green alga *Ulva conglobata*, Akakabe *et al.* [60] incubated crude extracts of this alga with arachidonic acid. Using a combination of HPLC-MS and headspace-SPME-GC-MS, they showed that 2,4-decadienals were produced via (*R*)-11-HPITE (hydroperoxyicosatetraenoic acid) from ARA exclusively. Compounds other than fatty acids have been described in green algae, thanks to the use of MS-based techniques. For instance, investigation of melatonin production as its TMS (TriMethylSilyl)-derivative by GC-MS allowed correlating accumulation of this compound with semi-lunar rhythm [61].

To close this section on macroalgae, it is interesting to note that there are a number of comparative analyses that have been performed to assess different metabolic abilities in seaweeds, in particular production of fatty acids, as illustrated by at least three publications in 2011. A first one analyzed the fatty acid composition of nine seaweed species from the North Sea and from tropical seas in the context of seaweeds being a good, durable, and virtually inexhaustible source of polyunsaturated fatty acids (PUFAs) [62]. Metabolites were analyzed as fatty acid methyl esters (FAMEs), and quantified by common GC-MS method. Among the results obtained, the n-3 docosahexaenoic acid (C22:6n-3, DHA) was only found in the brown alga *Sargassum natans*, while eicosapentaenoic acid (EPA, 20:5n-3) was found in the nine species tested, with the highest amount in the red alga *Palmaria palmata*. The same year, a similar study was also published [63], considering one species for each type of macroalgae tested, and aiming at evaluating and selecting a method for lipid and fatty acid extraction before running GC-MS. It was shown that the green algae *Ulva fasciata* was the only one to contain DHA. Moreover, the different extraction methods tested (e.g., Bligh and Dyer, Folch, and others) demonstrated that *U. fasciata* was a huge reservoir of fatty acids compared to red or brown seaweeds. Finally, a third comparative analysis, based this time on tropical seaweed from India, but focusing not only on determination of the fatty acid contents but also on mineral and antioxidant properties, has been published by the same research group [64], and confirmed the presence of DHA in all the green algae tested.

### 2.3. Animals (Vertebrates and Invertebrates)

Metabolomic data on marine animals have mostly been acquired by NMR [65,66], and this technology has also been combined with MS in a number of studies, representing a rather novel and complementary approach in the marine research area [67].

#### 2.3.1. Vertebrates

Karakash *et al.* [68] conducted a metabolomic study on salmon long-term handling stress by analyzing fish plasma through a combination of ^1^H NMR and LC-MS. The former technique indicated a change in the metabolic profile after one week of stress and for certain categories of compounds, while the latter technique showed difference mainly at week 2 of the treatment, and in relation to another set of compounds. Targeted metabolite profiling has also been described in fish, mainly for determination of contaminants in specific tissues. For instance, UPLC-MS-MS (in positive ESI mode) offers the possibility to quantify albendazole and its metabolites in 3 minute long runs [69]. Tandem MS was also used for the determination of benzotriazole ultraviolet stabilizers in fish [70]. Moreover, an LC-MS-MS-based lipidomics assay on *Engraulis* (Peruvian anchovy) [71] was applied to identify endogenous pro-resolving mediators such as resolvins, protectins and related compounds. More generally, in metabolomics science, lipidomics can be seen as targeted metabolite profiling since it concerns a specific class of compounds; meanwhile, the diversity of lipids, fatty acids, and their derivatives is so high that it needs several approaches if an exhaustive view of these molecules is required. As an example, using a Q-TOF method, Yuan *et al.* demonstrated that lipidomics is an effective analytical tool for predicting stress resistance of fish submitted to unpredictable environmental stress [72].

Targeted metabolomic studies have been conducted on other animals such as harbour seals and harbour porpoises [73,74]. Detection of a wide range of chlorinated and brominated contaminants was achieved by GC-MS in the EI mode and in the electron capture negative ion (ECNI) mode. Determination of limits of detection and of quantification demonstrates the ability of MS for quality/control applications.

#### 2.3.2. Invertebrates

Several types of mollusks have been considered recently for MS-metabolite profiling. One of the examples is the investigation of tidal cycle effects on mussel (*Mytilus californianus*), both by GC-MS and LC-MS (either in positive or negative mode), to obtain an exhaustive view of the metabolism related to this biological cycle [75]. This study allowed a survey of both hydrophilic and lipophilic metabolites in a large *m/z* range. In a different context, effects of zinc and cadmium exposure on clam metabolome have been described by coupling ^1^H NMR and GC-MS [76]. Beach *et al.* determined pyrene metabolites in marine snails, using LC-MS-MS in the multiple reaction monitoring (MRM) acquisition mode to lower the quantification limits by disregarding background noise [77].

Sponges are recognized as interesting sources of natural products with therapeutic applications, and many of the marine species host *cyanobacteria*, and less commonly Dinoflagellates. HPLC-diode array detector (DAD)-evaporative light scattering detector (ELSD)-MS has been used for metabolic fingerprinting dedicated to chemosystematics of marine organisms. For instance, the intra- and inter-specific metabolic variability of 10 Mediterranean *Homoscleromorpha* species was correlated with their bioactivity, endobiotic bacterial diversity, and mesophyll cell types [78]. MALDI-TOF imaging has been used to observe the spatial distribution of secondary metabolites within the sponge *Dysidea herbaceae* [28], and this approach has been shown to be of interest to study the biosynthetic origin of natural products identified from marine microorganisms-invertebrates (in particular marine sponges and ascidians) assemblages [79]. In another context, laser ablation electrospray ionization mass spectrometry (LAESI-MS), a quite uncommon MS technique, has been useful to work directly on samples such as sea urchin eggs, allowing detection of small metabolites along with lipids [80].

Corals, sessile animals among which most of them rely on photosynthetic microalgae (Zooxanthellae) to obtain the majority of their energy and nutrients, are a well-studied family of marine organisms because of their high content in natural products of pharmaceutical interest [81,82,83,84]. However, to our knowledge, no global metabolomic study has been published yet for these organisms. Nevertheless, MS has been widely used for targeted metabolite profiling such as determination of betaine metabolites, cembranolides, and for identification of chemicals of microbial origin in these invertebrates [85,86,87].

## 3. Metabolic Footprinting: Analysis of Seawater Using MS-Based Metabolite Profiling Techniques, a Powerful Tool in Chemical Ecology

It has been known for a long time that different types of living organisms secrete a large number of metabolites into their environment (especially under conditions of unbalanced growth) [88]. However, the external chemosphere of most marine organisms (including microorganisms) has remained overlooked until recent applications of MS-based chemical profiling and metabolomic approaches for chemical ecology studies. A statistical evaluation of data provides insights into the released metabolites that might represent a message sent by emitter organism(s) to potential receiver organism(s). Chemical cues and signals do not only drive predator/prey interactions; they affect critical processes such as mating and habitat choice, and they also produce a cascade of indirect effects that impact ecosystem functions [89]. Recent investigations in planktonic and benthic diatoms, and also in seaweed ecology, highlighted the resolving power of MS-based approaches.

An UPLC-MS approach, designed by Barofski *et al.*, showed the influence of *Skeletonema marinoi* growth phases on the cellular metabolic profile of a copepod, suggesting that changes in (info)chemicals within or surrounding the diatom regulate selective feeding of the zooplankton [90,91]. Also in diatoms, GC-MS analysis of ethylacetate extracts of cell-free spent culture medium, and of volatile organic compounds collected by headspace solid phase microextraction (HS-SPME), revealed the occurrence of 18 different brominated and iodinated volatiles in cultures of the biofilm-forming *N. *cf.* pellucida*, including the natural product cyanogen bromide (BrCN) which exhibits pronounced allelopathic activity [40]. It was shown that production of this toxic metabolite is light dependent with a short burst after sunrise. BrCN acts as a short-term signal, leading to daily “cleaning” events around the algae, and is dependent on haloperoxidase-mediated oxidation using hydrogen peroxide.

In macroalgae, a number of studies, conducted with ecologically relevant methodologies, have focused on secondary metabolites involved in the defense against biofoulers and pathogens [92]. More recently, in large brown seaweeds, the release of exudates was described as an important phenomenon mediating interactions of algae with either associated microorganisms or other interacting larger organisms. However, the chemical nature of released molecules remained nearly unknown, except for abundant volatile compounds such as organohalogens that were detected using GC-ECD (electron capture dissociation) profiling after trapping [50]. In kelps, an important breakthrough was the successful establishment of a protocol, using chemical derivatization and GC-MS analysis, to measure volatile short-chain fatty acids which are produced by lipid peroxidation in the first minutes following the simulation, using elicitors, of the perception of an attack by a pathogen or a grazer. This allowed measuring, for the first time from *L. digitata*, the liberation of aldehydes in both seawater and air [55]. These compounds are potential signals to warn kelps and activate transcriptional responses, as well as biosynthesis of potential toxic compounds against herbivores. Further results of metabolite profiling experiments have been combined with analyses of gene expression data to provide an integrative view of the responses of *L. digitata* in an ecological context [93]. Results obtained on the expression of elicitor-induced genes, combined to PUAs and VHOCs (volatile halogenated organic compounds) profiling by GC-MS, clearly demonstrated the existence of yet unidentified signaling cues between kelps that are reminiscent of the mechanism of defense priming in terrestrial plants [93].

In spite of these fascinating progresses in the understanding of novel chemical communications within communities of marine organisms, no global metabolomics study on environmental seawater samples has been published yet. So far, most results of targeted profiling have been obtained on supernatant of controlled cultures. It remains to be demonstrated that dilution effects occurring in field conditions do not preclude the detection of cues that are relevant in the context of shaping population and community structures. This work needs to use approaches without *a priori* on the chemical classes of involved metabolites. Some of the technical challenges that remain to be tackled are detailed in the following section, such as extraction of metabolites from large volumes of seawater using solid phase extraction, as previously used in diatom cultures [90]. For other examples in the field of plankton ecology and from the terrestrial field, excellent reviews have been published in recent years [94,95].

## 4. Technical Challenges

Developments of *in vitro* (wet) and *in silico* (dry) technologies are necessary for the production and exploitation of metabolite profiling data from marine systems. This can be achieved only by overcoming technical/methodological/management challenges, some of which are listed below.

### 4.1. Interferences

Salts, mainly NaCl, represent major interfering compounds. Moreover, salts polymerize in the ion source and create adducts which increase the difficulty of identifying molecular ions [96]. This is illustrated by analysis of metabolites extracted from seawater samples, such as UV filter compounds which present, in the ESI positive mode, similar or even more intense [M + Na]^+^ ions than the [M + H]^+^ [97]. In their publication, Nguyen *et al.* demonstrated that these [M + Na]^+^ ions were less reproducible and more resistant to fragmentation. As described by Shrestha & Vertes, dilution of samples was an imperative as high salt concentrations prevent metabolite detection [80].

Another challenge for metabolite extraction, in particular for macroalgal samples, is grinding. Indeed, seaweeds contain a lot of polysaccharides. For instance, in red algae, carrageenans confer a gelling nature to tissue, resulting in difficulties to obtain a reproducible manual grinding. Thus, particular attention has to be paid to grinding, and automated techniques to carry out such extraction step, e.g., cryo-grinding, should be preferred. To avoid metabolite degradation, freeze-drying of sample before grinding is an option; however, no study has been published yet on the comparison of metabolomic profiles of marine organisms obtained either by freeze-drying samples before grinding or by directly grinding samples in liquid nitrogen. Therefore, it is difficult to give any specific recommendations.

Other types of molecules, such as pigments, represent a major part of algal metabolite content [98]. Moreover, pigments have been reported to be major interfering compounds for MS analysis, as they induce source pollution and minimize ionization of other metabolites [99,100]. To prevent contamination, pigments can be removed by hexane/acetone extraction, as it has been described before for extracting phenols for analysis by LC-MS [101].

### 4.2. Extraction of Metabolites from Seawater

This type of experiment represents a great challenge, and we describe here different methods to extract chemicals from the seawater matrix.

Liquid/liquid extraction is one of the most current techniques to work on liquid samples. For instance, marine antifouling compounds organotins were extracted using liquid/liquid extraction, after derivatization, and then detected by either GC-MS or LC-APCI-MS (liquid chromatography-atmospheric pressure chemical ionization-mass spectrometry) [102]. However, liquid/liquid extraction is often time and solvent consuming. Therefore, solid phase extraction (SPE) appears to be a more adequate method for obtaining accurate and reproducible results. C18 SPE columns, based on the same principle of interactions like the ones used for HPLC, offer a wide extraction range, from hydrophilic to lipophilic compounds. Different volumes of sample, from 1 mL to 2 L, can easily be loaded, and then metabolites eluted with a small volume of solvent, typically 1 mL to 10 mL. SPE provides the possibility to fractionate the sample, separating metabolites with a higher affinity for chloroform from those with high affinity for methanol for instance. Furthermore, automated systems increase reproducibility, while decreasing operator actions and time required. As an example, Wu *et al.* proposed a SPE method for the determination of four pharmaceutical residues in seawater, and underlined the importance of adjusting deionized water volume to clean up the column from sodium chloride [103].

Substitutes to SPE exist, based on passive samplers such as polymethyldimethylsiloxane (PDMS). To quantify organotin in seawater samples, Chou *et al.* suggested sampling by headspace solid phase microextraction (HS-SPME) coupled with GC-MS [104]. In this study, organotins were derivatized with tetraethylborate and then heated for analytes to be adsorbed on the fiber, but this protocol induced degradation or oxidation processes. Moreover, the sampling time varied from 30 min to 1 h previous to injection, and one sample at a time, constraints which increased the complexity for automation and altered reproducibility. The HS-SPME-GC-MS method has also been used to demonstrate exudation of iodinated and brominated metabolites by a diatom [40], as described in the previous section. An alternative to SPME is stir bar sorptive extraction (SBSE). The sorbent is also PDMS. The main advantage of this process is that it is solvent free, and is therefore suitable for the detection of low mass metabolites, generally eluted in the solvent peak. It is possible to change the sorption equilibrium by modulating pH, temperature, or sodium chloride concentration. Thus, the high concentration of NaCl in seawater confers a higher extraction range on metabolites of low affinity for PDMS. Moreover, sample extractions can be carried out in parallel, thus limiting experimental variations. After stirring (from 1 h to overnight), metabolites can be desorbed either by thermo-desorption coupled with GC-MS, or by solvent extraction and then analyzed by LC-MS-MS. The latter technique was used by Nguyen *et al.* for the determination of UV filters in seawater [97]. In this study, performances of both atmospheric pressure chemical ionization (APCI) and ESI ionization were investigated, and APCI was selected for seawater analysis due to its higher sensitivity. SBSE is likely to be a suitable method for targeted metabolite analyses such as profiling volatile compounds. However, this extraction technique is not suitable for sulfated metabolites such as dimethylsulfide because they display a very low affinity for PDMS.

### 4.3. Data Treatment

**Figure 1:**
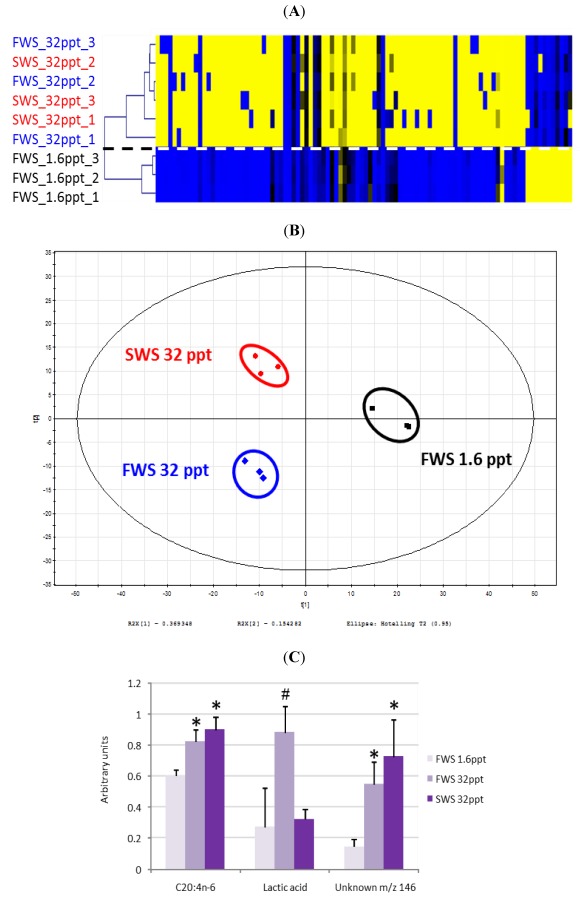
Metabolomic data obtained for two strains of *Ectocarpus* (brown alga) by Gas chromatography-mass spectrometry (GC-MS) analysis. Both strains corresponded to the reference genome-sequenced marine (SWS, CCAP 1310/4; [114]) grown in undiluted seawater (32 ppt) and a freshwater strain isolated from river falls in Australia (FWS, CCAP 1310/196; [115,116]) grown in undiluted (32 ppt) and highly diluted seawater (1.6 ppt), respectively. Algae were rinsed with distillated water before freezing and grinding in liquid nitrogen. Extractions were carried out with ethyl-acetate, and 12-hydroxy-lauric acid was added as internal standard. Metabolites were converted as Me-TMS-derivatives, and analyzed with an Agilent 7890 gas chromatograph equipped with a HP-5ms column (30 m, 0.25 mm internal diameter, 0.25 mm film thickness) coupled with a 5975 N mass spectrometer. Feature detection was done with AMDIS (version 2.1; Automated Mass Spectral Deconvolution and Identification System; National Insitute of Standards and Technology: Gaithersburg, MD, USA, 2006) [112], and comparative analysis with the online available SpectConnect [113]. All metabolites were considered for (**A**) Hierarchical tree clustering (*p*-value < 0.01) performed with TigrMeV 4.8; and (**B**) PLS-DA (*p*-value < 0.05) generated by SIMCA-P + 12.0 (Umetrics); (**C**) Relative abundance of arachidonic acid (ARA, C20:4n-6), lactic acid, and of an unknown compound with the more intense ion *m/z *146. Spectra obtained for the latter compound were compared in Golm metabolome database (GMD), Massbank, Human Metabolome Database (HMDB), and did not give any match allowing identification. * and # indicate that a mean differed significantly from the mean obtained for the samples FWS 1.6 ppt at *p* < 0.01 and *p* < 0.05 respectively.

Another challenge to conduct metabolomic studies is data treatment. For MS data, several pipelines are available such as XCMS [105], MetAlign [106], MZmine2 [107], MetaboAnalyst [108], metaP-server [109], MetAtt [110], Metabolome Express [111], AMDIS [112], or SpectConnect [113] (Table 1). These bioinformatics tools include several steps such as feature detection, grouping, retention time alignment, normalization, identification, and even statistical treatment. This last point represents a key aspect for metabolomic data interpretation since it implies not only the description of the metabolome of an organism, but also quantification and comparison of metabolite contents between several samples. The manner of processing these statistical analyses is crucial, since it determines the significant metabolites that can discriminate between two conditions. Numerous methods such as hierarchical clustering, principal components analysis (PCA), and partial least squares-discriminant analysis (PLS-DA) exist. The level of discrimination that can be obtained for each method may be different. An example of analysis of metabolic data in marine biological model is presented in Figure 1, and is based on a recent experiment performed in two strains of the brown alga *Ectocarpus*. Applying the method of hierarchical tree with a *p*-value < 0.01 to GC-MS does not allow to discriminate between the reference genome-sequenced marine strain (SWS) and a strain isolated from freshwater (FWS) when both are cultivated in undiluted seawater (32 ppt) (Figure 1A). However, separation is clear for the FWS grown in 32 ppt and 1.6 ppt conditions. PLS-DA conducted with a *p*-value < 0.05 clearly explains 37% of the variation with the first component, and about 15% variation with the second component (Figure 1B). Interestingly, and in contrast with the previous results, this analysis allows discriminating between both tested strains grown in undiluted seawater (32 ppt). Looking at the metabolites detected with this analysis, it is interesting to note that PLS-DA (*p* < 0.05) allows discrimination of more metabolites than hierarchical clustering (*p* < 0.01). As an example, lactic acid, which is not considered as a discriminant compound after hierarchical clustering, appears as one of the metabolites allowing discriminating SWS and FWS cultivated at 32 ppt after PLS-DA, through its contribution to the second component of this analysis (Figure 1C).

### 4.4. Towards Metabolomics Database Dedicated to Marine (eco)Systems

Identifying metabolites is a bottleneck in the metabolomic field. Wishard recently reported the complexity of identifying compounds (1000 to 200,000 different chemicals) when compared to the identification of genes and proteins [117]. In addition, as with any omics discipline, metabolomics is highly dependent on the availability and quality of a wide variety of electronic resources. Currently, there are at least five types of databases used in metabolomics research. These include: (i) metabolic pathway databases; (ii) compound-specific databases; (iii) spectral databases; (iv) disease/physiology databases for animal models; and (v) comprehensive, organism-specific metabolomic databases.

The KEGG and “Cyc” databases are examples of some of the most popular metabolic pathway databases. There is, therefore, a need to develop specific “Cyc” databases dedicated to some of the marine models for which genomes are sequenced (*Ectocarpus*, *Ostreococcus*), as recently done for the diatom *Phaeodactylum tricornutum* [118], and such as the ones listed within the BioCyc Database Collection (version 16.0, 16 February 2012) [119]. These resources contain carefully illustrated and hyperlinked metabolic pathways with synoptic metabolite information for a wide range of organisms. On the other hand, most of the compound-specific databases such as LIPID MAPS [120], KEGG Glycan [121], DrugBank [122], ChEBI [123], Chemspider [124], The Madison Metabolomics Consortium Database (MMCD) [125], and PubChem [126], do not contain pathway information. Instead, they focus on providing detailed nomenclature, structural, or physico-chemical data on restricted classes of compounds, such as lipids, carbohydrates, drugs, toxins, or other chemicals of biological interest. These somewhat specialized databases often contain metabolites or xenobiotics not found in most metabolic pathway databases. It will be essential to provide links to these databases in portals dedicated to marine resources. In addition, the democratization of precise mass determination instruments (FT-ICR, Orbitrap, TOF) has improved the ability of defining chemical formulas by MS techniques. However, MS identification always needs verification with standards. An alternative to standards is aligning/comparing spectra to commercial and open-sources databases. HMDB [127], Metlin [128], KNApSAcK [129], MassBank [130], FiehnLib [131], Golm metabolome database (GMD) [132], MMCD [125], NIST (National Institute of Standards and Technology, [133]), and LIPID MAPS [120] are powerful open-sources or commercial databases to compare mass spectrometry data to standards. However, most of the metabolites contained in these databases have been identified in land plants or humans.

Finally, it is also possible to suggest the building of organism-specific comprehensive metabolomic databases to combine metabolic information gained for specific marine organisms, and to link this type of repository to the other kinds of web resources listed above, to aim at knowledgebase(s) similar to the Human Metabolome Database (HMDB) [127].

Soon, the community of marine chemists, biologists, and ecologists will have the capacity to rapidly identify a large number of metabolites from various marine organisms or seawater samples using hyphenation of GC-MS and LC-MS. It will also be possible to identify metabolites without ambiguity using MS-MS. Details for the experimental procedures and analyses of data will be important to be recorded in the framework of the minimal requirements for publishing metabolite data [134]. In addition, editorial boards of dedicated marine journals would also have, ideally, to impose these important recommendations. Moreover, experimental standards and data exchangeability between laboratories and platforms using MS-based metabolomics will become an important issue for further developments in marine metabolomics. Future “marine” dedicated libraries will contain retention times, relative retention times (in reference to internal standards), and molecular formulas. For instance, it will be suitable to compile these libraries for species for which (large) genomics resources are available, and/or for species of special biological/ecological interest. As an example, a metabolite database on compounds extracted from macroalgae is available: the seaweed metabolite database (SWMD) indexes more than 500 metabolites mostly characterized from the red alga *Laurencia* [135]. In addition, the MarinLit database [136] mostly focuses on marine natural products; it contains bibliographic data and an extensive collection of keywords, trivial names, compound information including structures, formulae, molecular mass, numbers of various functional groups, and UV data. However, there is no possibility to compare/align mass spectra.

### 4.5. Federation of Marine Biologists and Ecologists for a Better Integration and Promotion of Standard Initiatives

**Figure 2:**
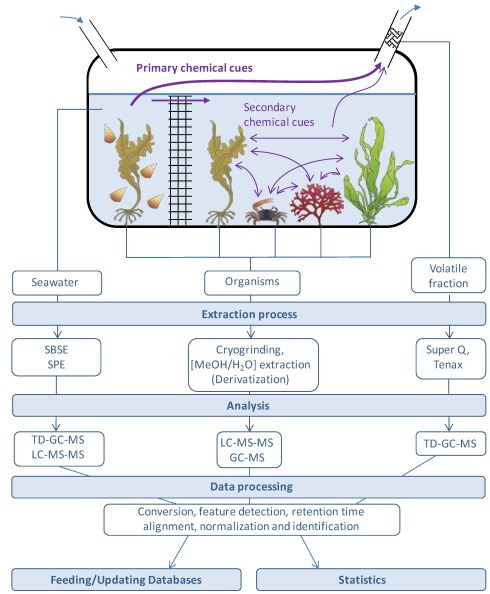
MetaboMER workflow for a metabolomic approach of marine ecology (adapted from [138]). We propose to create a marine biotope in the laboratory. On the left side of the net, a macroalga is submitted to herbivory through incubation in presence of marine grazers (e.g., snails, helcions). Other organisms are placed in the right compartment to test the existence and the impact of chemical cues. Phenomena such as priming, attraction of marine grazers predators, and metabolome modifications of macroalgae of the same species or of other species could be investigated. Kinetic studies may also be relevant to determine primary and secondary chemical cues. Symbols used for organisms are courtesy of the Integration and Application Network (http://ian.umces.edu/symbols/, accessed on 10 February 2012), University of Maryland Center for Environmental Science.

Quality control and standardization of metabolomics data are important technological points that are well considered in the biomedical and plant research fields, and are necessary to guarantee meaningful biological applications. Therefore, it is essential, in the nascent field of marine metabolomics, to anticipate these issues by increasing integration of platforms featuring different levels of specialization in some extraction procedures and MS-based approaches (Figure 2), in a network of more experimented sites involved in other applications of metabolomics, at national and international levels. Standard operation procedures for sample collection, homogenization, extraction and derivatization should be developed and optimized on the basis of already available methods. These protocols should be implemented in a local site and validated using the available metabolomics platforms of a larger network. Such a level of integration has been reached in the case of the platform that we have set up in our institute. The MetaboMER platform integrates the complete workflow from extraction of metabolites from organisms or biological fluids (including seawater samples), to analytical procedures using GC-MS, EI-MS, NICI-MS and LC-MS-MS. Metabolic profiles and target signatures are then handled by bioinformatic data management and beneficiate from expertise of our genomics center that helps to develop a central database and repository site. It also largely benefits from the Biogenouest^®^ bi-regional cluster that coordinates the life science core facility network in western France, and which include metabolomics facilities dedicated to the plant and medical fields. International efforts to coordinate actions of metabolomics platforms dedicated to marine biology will require the involvement of all countries with a strong vision of future of marine sciences. Future achievements will be implemented through research infrastructures within international consortia. Such an example of an integrative project is a consortium of seven of the main European marine biological stations promoting access to existing infrastructures via the Framework Programme 7 (FP7) Integrated Infrastructure Initiative (I3) project “Association of European Marine Biological Laboratories” (ASSEMBLE). In this network, two sites aim to provide access to facilities for marine metabolomics: the Station Biologique de Roscoff (CNRS-UPMC, France) and the Centre of Marine Chemical Ecology (Sven Lovén Centre for Marine Sciences of University of Gothenburg, Tjarno, Sweden). The next critical step is to develop a coherent pan-European infrastructure for provision of services related to marine genomics. This is the objective of the European Strategy Forum for Research Infrastructures (ESFRI) project “European Marine Biological Resource Centre” (EMBRC, [137]). The consortium involved in the preparatory phase of EMBRC comprises 12 key European marine biological laboratories, and it is expected that joint development actions will implement organism-centered and dedicated databases as described in the previous section.

## 5. Metabolite Profiling for Integrative and Systems Biology

Integration of data and systems biology approaches have already been applied to different marine systems, without any metabolite profiling data. Thus, different sorts of models are available for marine organisms, in particular for some selected organisms within their corresponding phylogenetic lineage. To study early development of the brown algal model *E. siliculosus*, Billoud *et al.* [139] developed a stochastic 1D nearest-neighbor automaton that was further implemented to characterize an artificial mutant altered in its pattern of development [140]. At the cellular scale, and for sea urchin, gene regulatory networks [141], and translational regulation networks [142] were developed a few years ago. Regarding metabolic processes, relevant networks have been reconstructed for marine microorganisms such as *Thermotoga maritima* [143], *Vibrio vulnificus* [144], and *Phaeodactylum tricornutum* [118]. Metabolic networks have also been reconstructed for fish using data from five genomes including marine species [145]. Mathematical modeling has been used recently to infer the dynamics of starch content during the diurnal cycle in the model green alga *Ostreococcus tauri* [146]. In addition, application of systems biology to brown algae, and in particular for *E. siliculosus*, has been highlighted to explore acclimation and adaptation to the intertidal zone [58].

Adding analysis at the level of metabolomes with other omics datasets (such as transcriptomes and proteomes to cite the most common) should help to go deeper in the understanding of the processes going on at the scale of an organism (cell, tissue, whole organism), and of the relationships within communities (population scale). Use of organisms considered as biological models within each of the phylogenetic lineages found in the seascape may be a suitable way to develop metabolomics approaches to study different aspects of biology, in particular specific adaptations, in relation with development and/or metabolic processes for instance. In order to integrate data produced at the transcriptome and the metabolome level, correlations between genes on one side and metabolites on the other side can be searched for. It is important to determine co-expression of genes and co-expression of metabolites separately, in order to group genes and metabolites according to similar patterns of expression or production, respectively. In this way, unknown genes will cluster with genes encoding proteins of known function (gene-gene correlations), and similarly, identified metabolites will group with metabolites of unknown structure (metabolite-metabolite correlations). It is possible to conduct similar types of analyses if proteome data are available for corresponding samples. Then it will be relevant to make the link between genes coding for well characterized proteins identified in gene expression clusters and well-defined metabolites contained in metabolite clusters, and carry on analysis in order to decipher metabolic processes. The co-occurrence of related transcripts and metabolites assessed by multivariate analysis may be a useful approach to infer functions of genes, not only in benchmark models, but also for non-model organisms. Effectively, it is currently possible to produce, quite readily, transcriptome data by NGS (Next Generation Sequencing) that can then be integrated with results corresponding to the full suite of metabolites measured in identical sample.

Transcriptome and metabolome data can also be used directly for reconstruction of metabolic networks. However, this type of reconstruction is so far mainly based on information from the sequencing of genomes. The establishment of connections between transcripts and metabolites, and integration of these data with previous genome data, can contribute to improve annotation, in particular for genomes of exotic organisms for which the number of new genes is high. It also permits to complete and check genome scale networks and to give some indications on potential regulation. This iterative process will help predicting behavior of models in order to make some hypotheses under different types of constraints and for several applications, such as the response to changes in environmental conditions. Predictions and hypotheses will need to be tested though measurement of reaction fluxes by fluxomics approaches such as stable isotope labeling techniques [147]. This additional layer of omics will give some indications on metabolic activity rather than focusing only on metabolite contents. It is based on analysis of mass isotopomers, and consists of feeding specific labeled metabolites (for instance, ^13^C or ^15^N, called isotopic tracers or isotope labeled precursors) to tissue or culture, and to assess the fate of these metabolites by MS-based techniques [148] or NMR [149]. To go further, the dynamic labeling approach enables the determination of metabolic fluxes from the kinetics of *in vivo* isotope labeling, and can allow investigating various aspects of metabolism, as it has already been the case in land plants [150], in particular for primary carbon metabolism [151]. Isotope labeling experiments are scarce in marine organisms using MS-based approaches, whereas important studies have been conducted, for instance in seaweeds, by pulse-chase labeling combined with NMR detection in order to investigate the flux of photosynthetic carbon. As an example, a recent study investigated the isotopomers composition and ^13^C-label distribution in low-molecular weight carbohydrates (floridoside and digeneaside) for the red alga *Solieria chordalis* incubated under different salinities [152].

Once pathways will have been reconstructed and analyzed, it will be possible to perform comparative and evolutionary analyses of metabolic processes among different lineages, to try to relate gain and loss of genes with different phenotypes and metabolic capabilities. This will be important to trace back the evolution of pathways, not only in marine organisms. However, in these organisms, it will help to understand why a lot of them exhibit so many peculiarities in metabolic processes, including at the level of primary and secondary metabolism. One step further will be to relate these specificities with their ability to thrive in their environment, considering also the diversity of ecosystems found in the seascape. In addition, to consider metabolic data for systems biology approaches in the framework of chemical ecology, and therefore to study biotic interactions between different types of organisms in a community, a higher level of integration will be necessary, for instance to observe how perturbations within metabolic networks of a given organism can alter the behavior of other metabolic networks contained in different organisms, belonging or not belonging to the same species.

## 6. Conclusion and Perspectives

In this review, we give an overview of recent literature published on metabolite profiling in marine systems. There is a wide and increasing variety of metabolomics applications in marine organisms, performed at different scales, either to discover molecules or to analyze a broad spectrum of metabolites; the latter can possibly be used for elucidating metabolic capabilities of a given species or metabolic responses triggered by changes in environmental conditions. Metabolites can exhibit high spatio-temporal heterogeneity, due in part to fine tuning of their production, in particular for regulatory molecules. In our opinion, one way of translating several pieces of literature leads us to say that monitoring changes in metabolite contents under variable growth conditions within an organism is like looking at the “genome in motion”. Effectively, metabolites present in a sample at a given time offer a valuable snapshot of what is happening at this time in a cell or in an organism if dealing with unicellular species. To produce this comprehensive coverage, there is a need for combining distinct analytical platforms based on different separation techniques coupled with MS or MS-MS. It may also be relevant, in some cases, to complete experiments with enzymatic determinations to obtain reliable and reproducible quantification of some metabolites.

Recent developments in metabolomics enable new approaches towards the characterization of the chemically mediated interactions of organisms with their environment. Particularly in chemical ecology research, where metabolic responses to stimuli like herbivory or mechanical tissue disruption are often relevant, metabolomic techniques have the potential to be an important complement to methods used traditionally [95]. By using metabolomic techniques, secondary metabolites produced or transformed during plant herbivore interactions can be recognized, and molecules expressed constitutively can easily be excluded for further analysis. Thereby, the task of identifying relevant metabolites in e.g. defense responses is made easier.

Finally, technical developments will be welcome, in particular for fluxomics, to monitor metabolites in a dynamic way, and for MS-imaging, to precisely localize and quantify metabolites in cells and tissues. This will strengthen applications of metabolomics in systems biology approaches to study unique metabolic pathways present in marine organisms and their regulation, and also to analyze what the metabolites are and how they act to mediate biotic interactions in marine environments. Metabolomics should also be a valuable contribution to the growing interest in marine biotechnology by providing further applications in the context of green and blue chemistry.

## Acknowledgments

We would like to dedicate this publication to the memory of our friend Jean-Pierre Salaün, who untimely passed away on 22 June 2011. His enthusiasm and inspiration for science—in particular, oxylipin biochemistry—will be unforgotten.

This work is a contribution within the project IDEALG which benefited from the support of the French Government run by the National Research Agency and with regards to stimuli program entitled “Investissements d’avenir, Biotechnologies-Bioressources”. The set-up of the MetaboMER platform was supported by the European Regional Development Fund (ERDF)-FEDER 33765, Région Bretagne, French Ministry for Research, UPMC, CNRS, and GIS Biogenouest.
